# A multidisciplinary approach to management of a non‐traumatic subdural hematoma in the third trimester of pregnancy: A case report and review of literature

**DOI:** 10.1002/ccr3.8510

**Published:** 2024-02-06

**Authors:** John Lugata, Caleigh Smith, Onesmo Mrosso, Tecla lyamuya, Bariki Mchome, Patricia Swai

**Affiliations:** ^1^ Department of Obstetrics and Gynecology Kilimanjaro Christian Medical Center Moshi Tanzania; ^2^ Kilimanjaro Christian Medical University College Moshi Tanzania; ^3^ University of Virginia School of Medicine Charlottesville Virginia USA

**Keywords:** Burr hole, cesarean section, multidisciplinary, non‐traumatic, subdural hematoma

## Abstract

**Key Message:**

Although non‐traumatic SDHs are uncommon during and immediately following pregnancy, management of these cases should be carried out by a multidisciplinary team, including obstetricians, pediatricians, neurosurgeons, and anesthesiologists.

**Abstract:**

Intracranial hemorrhage represents an uncommon but serious complication of pregnancy. Non‐traumatic subdural hematomas (SDH) are uncommon during the prenatal period with limited literature about etiology and management. In this case report, the authors report on a patient with non‐traumatic SDH in the third trimester of pregnancy. The patient is a 40‐year‐old G6P5L5 female at gestational age of 34 weeks and 5 days presenting with frontal headache, nausea, vomiting, and blurry vision. CT scan revealed an acute on chronic right subdural hematoma with midline shift and multiple herniations. An emergency cesarean section and right burr hole SDH evacuation were performed. Etiology of the SDH remains unknown. Although non‐traumatic SDHs are uncommon during and immediately following pregnancy, health care providers should always consider this possibility if a patient presents with typical symptoms and signs. Management of these cases should be carried out by a multidisciplinary team, including obstetricians, pediatricians, neurosurgeons, and anesthesiologists to optimize maternal and fetal outcomes.

## 1 INTRODUCTION

While intracranial hemorrhage is uncommon during pregnancy, it is a severe and sometimes fatal complication that contributes to maternal mortality and morbidity around the world. The most likely etiologies are ruptured aneurysms, hypertension, and trauma.^1^ When it comes to subdural hematomas (SDH) in particular, these are most commonly due to trauma causing rupture of the bridging veins, though the incidence of such injuries in pregnancy remains low.

Non‐traumatic, or spontaneous, SDH during pregnancy is even rarer with the relevant literature being limited primarily to case reports. Previously described causes of spontaneous prenatal SDH include ruptured aneurysms, preeclampsia, arteriovenous malformations, cocaine use, and coagulopathy, with pregnancy‐associated hypertension and preeclampsia appearing to be the most common contributing factors.^1^, ^2^, ^3^ In this case report, the authors report on a patient with non‐traumatic SDH of unknown origin in the third trimester of pregnancy.

## 2 CASE HISTORY

A 40‐year‐old female G6P5L5 presented to our specialty hospital center in Northern Tanzania at 34 weeks and 5 days gestational age with a chief complaint of severe frontal headache. The patient presented initially to a regional hospital which referred her to our center for specialty care. Her symptoms had been worsening over 10 days, and the headache was associated with projectile vomiting, nausea, and blurred vision. She denied any history of convulsions, loss of consciousness, and recent falls or trauma. She had regular antenatal follow‐ups, which had been uneventful with consistently normal blood pressure. Their past medical history was unremarkable, and she had no history of alcohol or cigarette use.

On physical examination, the patient was visibly uncomfortable but was alert and oriented with a Glasgow Coma Scale score of 15. Vital signs were stable, with a normal blood pressure of 114/64 mmHg, pulse of 82 beats per minute, and mildly elevated respiratory rate of 23 breaths per minute. Neurologic examination was unremarkable and nonfocal. Abdominal examination revealed a non‐tender, gravid abdomen and fundal height of 34 cm, consistent with gestational age.

## 3 METHODS

Leading differential diagnoses at this time included traumatic or non‐traumatic brain injury resulting in subarachnoid, subdural, or epidural hemorrhage, cerebral malignancy, pre‐eclampsia, and ischemic stroke.

Routine laboratory investigations were performed, all of which were within normal limits. Of note, her hemoglobin was 11.2 g/dL, platelets were 193 L, international normalized ratio (INR) was 0.93 s, and partial thromboplastin time (PTT) was 24.1 s. Obstetric ultrasound confirmed a single fetus with an estimated age of 36 weeks and 5 days and a weight of 3 kg. Fetal heart rate was 140 beats per minute, breathing and corporeal movements were visible, and fluid levels were appropriate.

**FIGURE 1:**
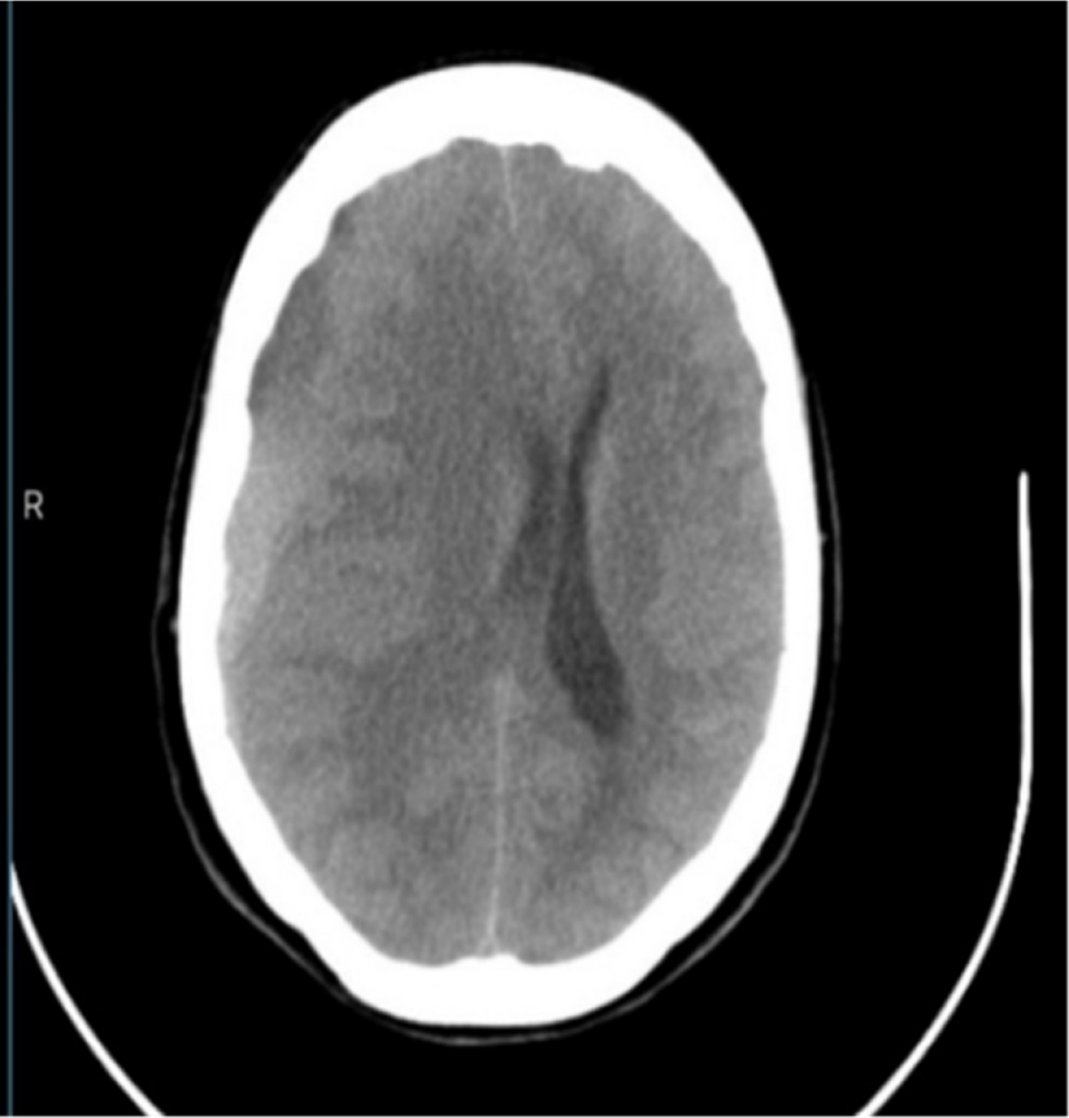
Computed tomography axial image showing acute on chronic right frontal parietal subdural hematoma measures 27 cm^3^ with midline shift of 1.7 cm with subfalcine herniation, ipsilateral trans tentorial, uncal hernation, and cerebellar tonsillar herniation by 5 mm. Positive for generalized cerebral edema and mild left obstructive ventriculomegaly.

**FIGURE 2:**
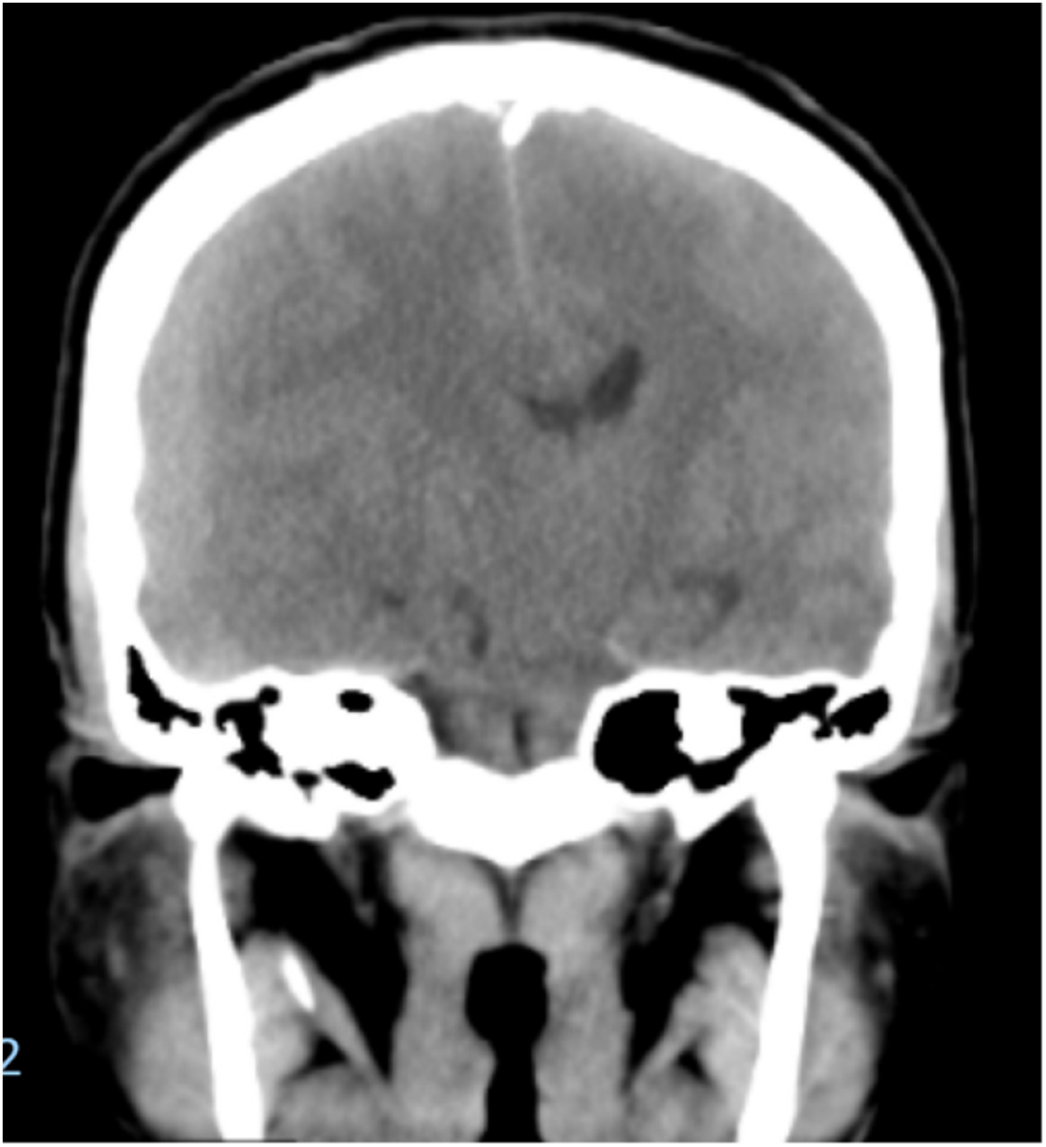
Computed tomography coronal image showing right subdural hematoma with midline shift.

Given the severity of the patient's symptoms, a non‐contrast head computed tomography (CT) scan was immediately performed and revealed an acute chronic right frontal‐parietal subdural hematoma measuring 27 cm^3^ (Figures 1 and 2). There was a significant midline shift of 1.7 cm with subfalcine herniation, ipsilateral transtentorial herniation, uncal herniation, and cerebellar tonsillar herniation by 5 mm. CT was also notable for generalized cerebral edema and mild left obstructive ventriculomegaly. Of note, we recognize that magnetic resonance imaging (MRI) may have been a useful alternative imaging modality, however pursuing an MRI is cost prohibitive for most of our patients in Northern Tanzania. This patient came from a poor rural community, so we discussed the utility and costs of both imaging approaches and decided to get a CT based on this shared decision‐making approach.

Immediate consultations were made for neurosurgery and anesthesia to plan for urgent surgical intervention. Pediatrics was also consulted for premature delivery planning. Upon coordination with the teams and discussion with the patient, the patient was quickly transferred to the main operating room for a simultaneous emergency cesarean section and right burr hole procedure to evacuate the hematoma. The cesarean section with an elective bilateral tubal ligation was performed by the obstetrics team without complications. The patient successfully delivered a male infant with a birth weight of 2.9 kg and APGARS of 8 and 10 at 1 and 5 min respectively. The neurosurgery team simultaneously performed a burr hole operation via linear scalp incision around the right parietal area. One burr‐hole was made, the dura layer was opened in a cruciate fashion, and approximately 60 mLs of hemolyzed blood was drained from the subdural space. A subdural drain was inserted and maintained post‐operatively as well.

Postoperatively, the patient was transferred to the surgical intensive care unit (SICU) while the newborn was admitted to the neonatal intensive care unit (NICU) for observation. The newborn transitioned well and was discharged from the NICU to the mother's room once she was stable.

## 4 CONCLUSION AND RESULTS

Postoperative management of the mother focused on pain management and frequent neurological exams to monitor her status. She received intravenous ceftriaxone and metronidazole for 3 days following surgery. On postoperative day (POD) 3, the patient was transferred from the SICU to the general obstetrics unit where she was managed primarily by the obstetric team with daily visits by neurosurgery as well. On POD 6, the patient was doing well and was deemed stable for discharge.

She returned to the clinic on POD 14 for follow‐up appointments with the obstetrics, neurology, and pediatrics teams. She was doing well without complaints at this time, and her cranial and abdominal stitches were both removed without issue. Based on her clinical stability and normal examination, neurosurgery decided to forgo repeat head imaging. Additional imaging such as a repeat CT or angiography would also have been cost‐prohibitive for this patient. She will return for monthly follow‐up appointments with neurosurgery for an indefinite duration.

## 5 DISCUSSION

Here we present a case of non‐traumatic SDH in a third‐trimester pregnant female. While overall uncommon, when traumatic SDHs do occur in pregnancy they tend to happen most commonly around the 33rd week of pregnancy, similarly to our case.^4^ Our patient presented with typical signs and symptoms of a subdural hematoma including headache, nausea, vomiting, and blurred vision. In other cases, patients may present with focal neurological symptoms, reduced consciousness, and seizures.^5^

In our case, the patient's initial symptoms of headache, nausea, vomiting, and blurred vision were highly suspicious for preeclampsia on admission, but we were subsequently reassured by her normal vital signs and laboratory tests. Thus, our remaining differential diagnosis included migraine, space‐occupying lesion, intracranial hemorrhage, and infection, prompting a CT to evaluate life‐threatening intracranial etiologies. Despite this patient's intact neurological exam, the CT revealed a significant SDH with minor herniation, suggesting a potentially devastating injury. Of note, our patient's SDH cannot necessarily be considered spontaneous given her imaging findings that show an acute on chronic SDH suggesting that her presenting symptoms may be due to re‐bleed.

The exact cause of the current patient's SDH remains unknown. She denied any recent trauma and did not have any apparent risk factors to explain the bleeding. Based on limited case reports, many non‐traumatic SDHs during pregnancy occur in the setting of hypertension with or without preeclampsia.^1^, ^2^, ^4^ In these cases, increased intracranial pressure as well as associated thrombocytopenia or coagulation abnormalities may contribute to bleeding risk. In addition, pregnancy is associated with physiological hormonally mediated changes in circulation, vascular tissue structure, and coagulability, all of which can contribute to further increased risk of stroke and bleeds.^6^ Of course, SDH in pregnancy may be due to classical causes as in a non‐pregnant patient such as aneurysm ruptures or AVMs which could be visualized via angiography.

Regardless of etiology, management of SDH in pregnancy is particularly complex, and there are no evidence‐based guidelines given the rarity of this condition. Approaches for diagnosis and management are limited by maternal and fetal safety concerns such as risk of radiation exposure, medication use, and surgical intervention. Of note, the hesitancy of neuroimaging during pregnancy is not warranted and the benefits of diagnosis outweigh any real or theoretical risks of radiation to mom or fetus. Regardless, symptomatic SDH requires urgent intervention to reduce intracranial pressure (ICP) and ensure stabilization of the patient which can be accomplished by medical treatment or surgical intervention or a combination of both. For stable patients with subacute or chronic SDHs, conservative treatment may be a viable option, particularly if there is no brain herniation or brain stem compression.^5^ In pregnancy, some commonly used medications may also be contraindicated, further necessitating the utility of co‐management by the obstetrical and neurosurgical teams. In unstable patients, patients with ongoing bleeding, or any signs of increased ICP necessitate prompt surgical evacuation. Such procedures are certainly possible and warranted in pregnancy but are again complicated by restricted medications, anesthesia challenges, and heightened risks to both the mother and infant.^7^ At our hospital in Northern Tanzania, we are unable to pursue advanced imaging or complex surgical approaches due to financial and technological limitations in this low‐resource setting. Variable resource availability is crucial to consider when designing optimal treatment plans for our patients, and each approach is individualized.

Non‐traumatic SDHs are uncommon during pregnancy and post‐delivery, but health care providers should always consider such a possibility if a patient presents with typical symptoms and signs. Management should be individualized and ideally managed in specialty referral centers with a multidisciplinary team, including obstetricians, pediatricians, neurosurgeons, and anesthesiologists, to ensure the best possible for both the mother and the fetus.

## AUTHOR CONTRIBUTIONS

**John Lugata:** Conceptualization; data curation; investigation; methodology; writing – original draft. **Caleigh Smith:** Conceptualization; data curation; investigation; methodology; writing – original draft. **Onesmo Mrosso:** Conceptualization; investigation; methodology; writing – original draft. **Tecla Lyamuya:** Conceptualization; data curation; investigation; methodology; writing – original draft. **Bariki Mchome:** Investigation; methodology; supervision; writing – review and editing. **Patricia Swai:** Investigation; methodology; supervision; writing – review and editing.

## FUNDING INFORMATION

This work did not receive any funds from any source.

## CONFLICT OF INTEREST STATEMENT

All authors have declared that no competing interests exist.

## ETHICAL CONSIDERATION

The patient provided written informed consent to allow for her de‐identified medical information to be used in this publication. A waiver for ethical approval was obtained from the authors' institution review board committee.

## CONSENT

Written informed consent was obtained from the patient to publish this report in accordance with the journal's patient consent policy.

## Data Availability

There is no data generated from this study.

## References

[1] Oudghiri N , Behat M , Elchhab N , Doumiri M , Tazi AS . Spontaneous subdural hematoma associated with preeclampsia: a case report and literature review. Pan Afr Med J. 2014;19:213. doi:10.11604/pamj.2014.19.213.5451 25829978 PMC4372310

[2] Giannina G , Smith D , Belfort MA , Moise KJ . Atraumatic subdural hematoma associated with pre‐eclampsia. J Matern Fetal Med. 1997;6(2):93‐95. doi:10.1002/(SICI)1520-6661(199703/04)6:2<93::AID-MFM5>3.0.CO;2-K 9086423

[3] Traficante DC , Marin A , Catapano A . Atraumatic subdural hematoma in a third‐trimester gravid patient. Case Rep Emerg Med. 2016;2016:8252746. doi:10.1155/2016/8252746 26989526 PMC4773536

[4] Ascanio LC , Maragkos GA , Young BC , Boone MD , Kasper EM . Spontaneous intracranial hemorrhage in pregnancy: a systematic review of the literature. Neurocrit Care. 2019;30(1):5‐15. doi:10.1007/s12028-018-0501-4 29476390

[5] Pierre L , Kondamudi NP . Subdural hematoma. StatPearls. StatPearls Publishing; 2023. http://www.ncbi.nlm.nih.gov/books/NBK532970/ 30422565

[6] Tate J , Bushnell C . Pregnancy and stroke risk in women. Womens Health. 2011;7(3):363‐374. doi:10.2217/whe.11.19 PMC3137888 21612356

[7] Upadya M , Saneesh P . Anaesthesia for non‐obstetric surgery during pregnancy. Indian J Anaesth. 2016;60(4):234‐241. doi:10.4103/0019-5049.179445 27141105 PMC4840802

